# Copy number variants and fixed duplications among 198 rhesus macaques (*Macaca mulatta*)

**DOI:** 10.1371/journal.pgen.1008742

**Published:** 2020-05-11

**Authors:** Marina Brasó-Vives, Inna S. Povolotskaya, Diego A. Hartasánchez, Xavier Farré, Marcos Fernandez-Callejo, Muthuswamy Raveendran, R. Alan Harris, Douglas L. Rosene, Belen Lorente-Galdos, Arcadi Navarro, Tomas Marques-Bonet, Jeffrey Rogers, David Juan

**Affiliations:** 1 Institut de Biologia Evolutiva (CSIC-Universitat Pompeu Fabra), Parc de Recerca Biomèdica de Barcelona, Barcelona, Catalonia, Spain; 2 Laboratoire de Biométrie et Biologie Évolutive UMR 5558, Université de Lyon, Université Lyon 1, CNRS, Villeurbanne, France; 3 Veltischev Research and Clinical Institute for Pediatrics of the Pirogov Russian National Research Medical University, Moscow, Russia; 4 National Centre for Genomic Analysis-Centre for Genomic Regulation, Barcelona Institute of Science and Technology, Barcelona, Catalonia, Spain; 5 Human Genome Sequencing Center, Baylor College of Medicine, Houston, Texas, United States of America; 6 Department of Molecular and Human Genetics, Baylor College of Medicine, Houston, Texas, United States of America; 7 Department of Anatomy and Neurobiology, Boston University School of Medicine, Boston, Massachusetts, United States of America; 8 Department of Neuroscience, Yale School of Medicine, New Haven, Connecticut, United States of America; 9 National Institute for Bioinformatics (INB), Barcelona, Catalonia, Spain; 10 Institució Catalana de Recerca i Estudis Avançats, Barcelona, Catalonia, Spain; 11 Institut Català de Paleontologia Miquel Crusafont, Universitat Autònoma de Barcelona, Cerdanyola del Vallès, Catalonia, Spain; Oregon Health and Science University, UNITED STATES

## Abstract

The rhesus macaque is an abundant species of Old World monkeys and a valuable model organism for biomedical research due to its close phylogenetic relationship to humans. Copy number variation is one of the main sources of genomic diversity within and between species and a widely recognized cause of inter-individual differences in disease risk. However, copy number differences among rhesus macaques and between the human and macaque genomes, as well as the relevance of this diversity to research involving this nonhuman primate, remain understudied. Here we present a high-resolution map of sequence copy number for the rhesus macaque genome constructed from a dataset of 198 individuals. Our results show that about one-eighth of the rhesus macaque reference genome is composed of recently duplicated regions, either copy number variable regions or fixed duplications. Comparison with human genomic copy number maps based on previously published data shows that, despite overall similarities in the genome-wide distribution of these regions, there are specific differences at the chromosome level. Some of these create differences in the copy number profile between human disease genes and their rhesus macaque orthologs. Our results highlight the importance of addressing the number of copies of target genes in the design of experiments and cautions against human-centered assumptions in research conducted with model organisms. Overall, we present a genome-wide copy number map from a large sample of rhesus macaque individuals representing an important novel contribution concerning the evolution of copy number in primate genomes.

## Author summary

One commonly used model organism in biomedical research is a species of Old World monkey, the rhesus macaque (*Macaca mulatta*). When translating biomedical research findings from rhesus macaques to humans, knowledge about the genetic differences between these two species is fundamental. In this study, we focus on copy number variation, which happens as a consequence of large duplication and deletion events in the genome, and which often has been overlooked. Copy number variation is a type of genetic variation which consists of one individual having more copies of a particular genomic region than another individual. We have performed an extensive evaluation of the copy number variants and fixed duplications among the genomes of 198 rhesus macaques and have generated a copy number map of high resolution for this species. We find that the overall pattern of within-species variation is similar to that in humans. However, when comparing specific duplicated regions between rhesus macaques and humans, we do find differences that could potentially have functional consequences. We have identified genes associated with human disease that have different copy number profiles between these two species, and therefore, we suggest avoiding human-centered assumptions in biomedical research conducted with model organisms.

## Introduction

Copy number differences are one of the fundamental aspects of genetic diversity within and between species. They can account for significant morphological [1] or physiological [2] differences among species and even result in the origin of new genomic functions [3]. This is especially true when copy number differences involve functional genes [4]. Among primates, comparisons of reference genome sequences have identified differences in gene copy number between humans and our closest relatives, the great apes [5–7]. Greater differences have been found relative to other more distantly related primates [8–12]. Within human populations, copy number differences have been associated with variation in gene expression [13] and differential risk for a wide variety of diseases [14], including autism [15] and other neurodevelopmental disorders [16], epilepsy [17] and infectious diseases [18]. A more complete understanding of copy number variation within and between various species will improve our understanding of both the potential clinical effects of human copy number mutations and the mechanisms and long-term patterns of genome adaptation and evolution.

Rhesus macaques (*Macaca mulatta*), a species of Old World monkeys that diverged from the superfamily Hominoidea (which includes gibbons, great apes, and humans) about 26 million years ago [19], are the most widely used nonhuman primate in biomedical research [20]. Through decades, researchers have conducted extensive analyses of the neuroanatomy and neurobiology of this species [21–24], including studies of gene expression during neurodevelopment [25]. Moreover, this species has been critically important in the study of infectious diseases [26–29] and as a model for other aspects of human health and physiology [20].

Although rhesus macaques have the widest geographic distribution of any nonhuman primate, stretching from Pakistan in the west to the Pacific coast of China in the east [30], the majority of rhesus macaques in North American research laboratories are of Indian origin. Recent studies have shown that Indian-origin rhesus have a large effective population size and higher levels of single nucleotide variation than humans [31,32]. In addition, the reference genome for this species contains numerous segmental duplications [9,33–35]. Together, these observations imply that rhesus macaques should, like humans, exhibit a substantial amount of intra-specific gene copy number variation.

In 2008, Lee and collaborators [36] examined a small number of macaques using microarray-based comparative genomic hybridization (aCGH) and found clear evidence for copy number variation in macaques, including some genes associated with human disease. Subsequent analyses have shown that particular gene copy number variants among rhesus macaques can influence phenotypes relevant to human disease [37–42]. For example, differences in progression rates to simian-AIDS have been observed between Chinese- and Indian-origin macaques [36]. This effect has been associated with differences in copy number of *CCL3-like* genes, which are a known host factor for HIV susceptibility in humans [37]. Despite these important observations, all previous studies of copy number variation in rhesus macaque were limited either in the breadth of genes investigated or the number of macaque individuals tested, or both.

Here we present the first genome-wide map of the copy number architecture and variation identified across a large panel of 198 rhesus macaque genomes. We report specific protein-coding genes that display copy number variation among this sample of rhesus macaques. We also identify genes that differ in copy number variability between rhesus macaques and humans. Each of these cases is relevant for understanding molecular similarities and differences between humans and other primates, and therefore for improving the use of rhesus macaques as genetic models of both normal human biology and its dysfunction. This knowledge will help to define the advantages and limitations of using this species as a model organism and refine the interpretation of macaque genetic data.

## Results

We generated a fine-scale genome-wide map of copy number for 198 rhesus macaques sequenced at high coverage using sequencing reads mapped to the Mmul_8.0.1 reference. This panel of samples was obtained after applying a very stringent quality-control filtering to whole genome sequences from an initial set of 315 samples integrating 213 samples from Xue et al. [31] and 202 newly sampled individuals. After quality control, we retained 51 and 147 samples (average coverage ~35x and ~32x respectively), respectively, forming a total of 198 samples (see Methods for details regarding the quality control pipeline; S1 Table).

**Fig 1:**
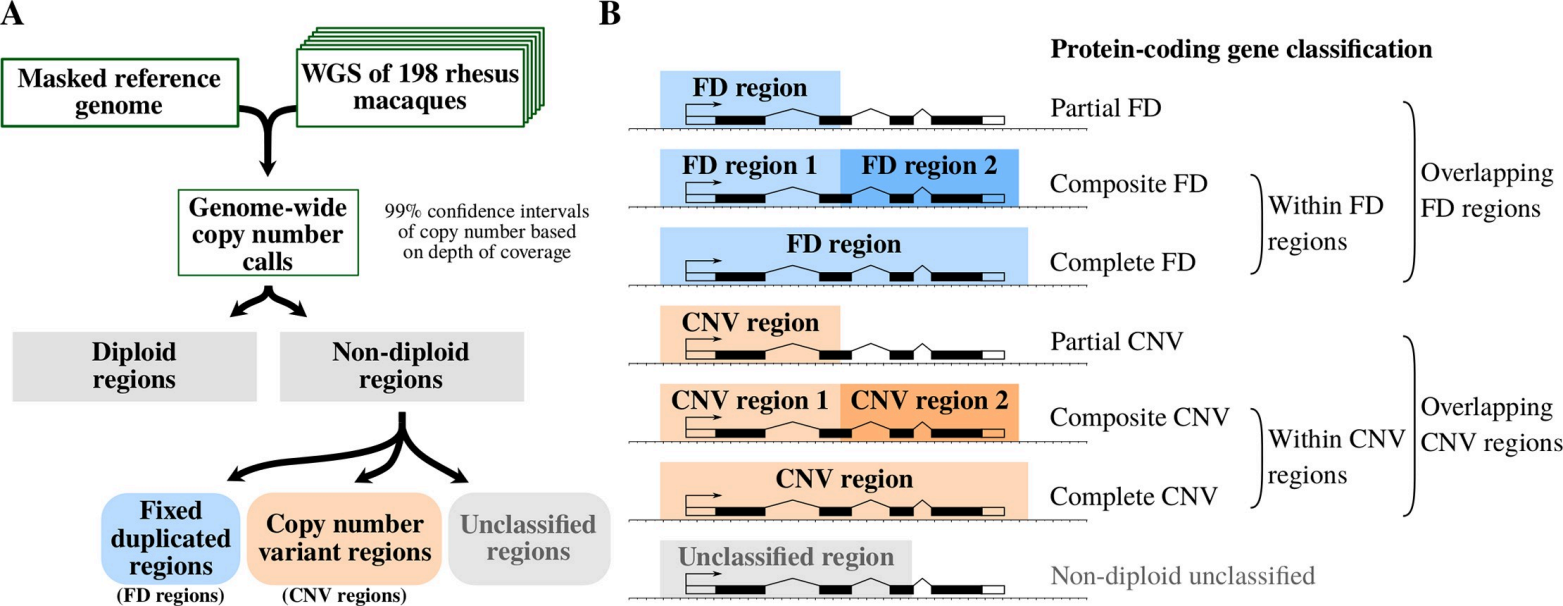
Summary of the methodology and protein-coding gene classification. (A) Starting with WGS data and a masked genome reference, we followed a novel approach in order to call copy number intervals for each non-repetitive 1 kbp window and sample based on depth of coverage. We performed a copy number allele calling per window to classify all non-diploid windows in *fixed duplicated regions* (FD regions), *copy number variant regions* (CNV regions), or *unclassified non-diploid regions*. (B) To assess the functional implications of non-repetitive windows, we crossed the protein-coding genes in the corresponding genome with the three different types of non-diploid regions. From all the genes that were related to FD or CNV regions, we distinguished between: *partial FD genes* or *partial CNV genes*, whose length is partially covered by an FD or a CNV region; *composite FD genes* or *composite CNV genes* which are within several FD or CNV regions (with 90% or more of their coding length covered by several FD or CNV regions); and *complete FD genes* or *complete CNV genes* which are within a single FD or CNV region (i.e., they show a constant copy number pattern per sample along all their coding length). All the protein-coding genes with the coding sequence partially or totally covered by non-diploid unclassified regions were classified as non-diploid unclassified genes. See S1 Fig and S5 Fig, and Methods for details.

To generate our copy number map, we identified non-diploid regions in all 198 rhesus macaque genomes. For every sample, we compared the depth of coverage of each non-repetitive 1 kbp window to those of a set of diploid regions (see Methods; S2 Table) through a novel statistical approach [43] (Fig 1A and S1A Fig; see Methods for details). This method has already been used in several publications [43–47]. As other methods based on raw read depth, our approach requires high-quality, high-coverage genome-wide sequence data in order to have a high signal-to-noise ratio [48]. Importantly, provided this requirement, copy number estimations by this method do not depend on assembly quality since the number of reads mapping to a specific region will remain proportional to the number of copies of the latter regardless of whether they are resolved in the reference genome or not [33,49]. However, the number of regions within the assembly that are detected to be duplicated or copy number variable by our method will depend on the assembly quality.

For example, consider we are analyzing the copy number of a given duplicated region in one individual. Imagine two possible scenarios. In the first scenario, both copies of this region are present (resolved) in the reference assembly. In this case, our method will identify both regions with copy number 4, since reads from both copies will be mapped to both regions. In the second scenario, only one of the two copies is present in the reference assembly of the species (the duplication is unresolved in the assembly). In this case, our approach will correctly identify the copy present in the reference with copy number 4 (reads from both copies will be mapped to it), but will not recover the copy missing in the assembly. The balance between both scenarios varies with the quality of reference genome assembly of the species, leading to an underestimation of the total number of duplicated regions in species with lower quality assemblies (two regions in the first scenario versus only one region in the second scenario). Crucial for our analyses is that our approach reliably determines copy numbers for any region present at least once in the assembly, allowing a robust comparison between orthologous regions, regardless of the differences in the qualities of the genome assemblies of the compared species.

Hence, our method based on read depth is extremely powerful to detect fixed duplications and copy number variants for cases in which at least one of the copies is present in the assembly. That is, we can detect fixed duplications and copy number variants in all the regions in the assembly. However, given that complex duplicated regions are among the most difficult to resolve [50] and the lower quality of the rhesus assembly (especially compared to the current human assembly), the number of duplicated and copy number variant regions identified within the rhesus macaque assembly must be considered as underestimates of the actual number of duplicated and copy number regions.

Genome-wide comparisons between human and macaque maps are challenging not only due to differences in assembly qualities but also because of the intrinsic differences in population structure and global species genomic variability. To establish a human map suitable for detecting copy number differences between humans and rhesus macaques, we applied the same algorithm we used for the macaques to a set of 32 human samples (50 samples before quality control; see Methods) with high-coverage whole-genome sequence data from the Simons Genome Diversity Project [51]. The individuals in this sample were selected as a diverse representation of human populations, providing an ideal dataset for retrieving inter-population copy number variability. This sample selection strategy enables us to detect differences in rhesus copy number associated with the most relevant human copy number variable regions. Although many more samples are available for humans, our strategy aims to reduce the influence of very low frequency inconsequential human CNV alleles while focusing on high-quality high-coverage sequence data.

### High-quality genome-wide maps of fixed duplicated regions and copy number variant regions

We expect, *a priori*, for most regions of the reference to be diploid, that is, to have two copies (one in each chromosome) in any given genome. However, read depth in the genome can vary due to a wide variety of reasons. We have accounted for intra- and inter-sample read-depth variability in order to distinguish between diploid and non-diploid regions, with the latter being a consequence of duplications and/or deletions. Among the non-diploid regions, we confidently distinguished between *fixed duplicated regions (FD regions)* and *copy number variant regions (CNV regions)*. FD regions are regions with the same non-diploid copy number in all the individuals of our sample.

On the other hand, CNV regions are those in which we could distinguish, with high confidence, more than one copy number allele within our sample. CNV regions include regions with gains, losses, and gains/losses of copies. Non-diploid regions with uncertain copy number distribution were labeled as *unclassified regions*. Typically, the unclassified regions contain different alleles with a similar copy number that could not be distinguished from inter-sample noise at our stringent confidence level. Accordingly, the unclassified category most likely contains regions that are actual CNV regions, but that we could not confidently sort into two or more clearly distinguishable copy number alleles (Fig 1A and S1A Fig; see Methods for details).

**Fig 2:**
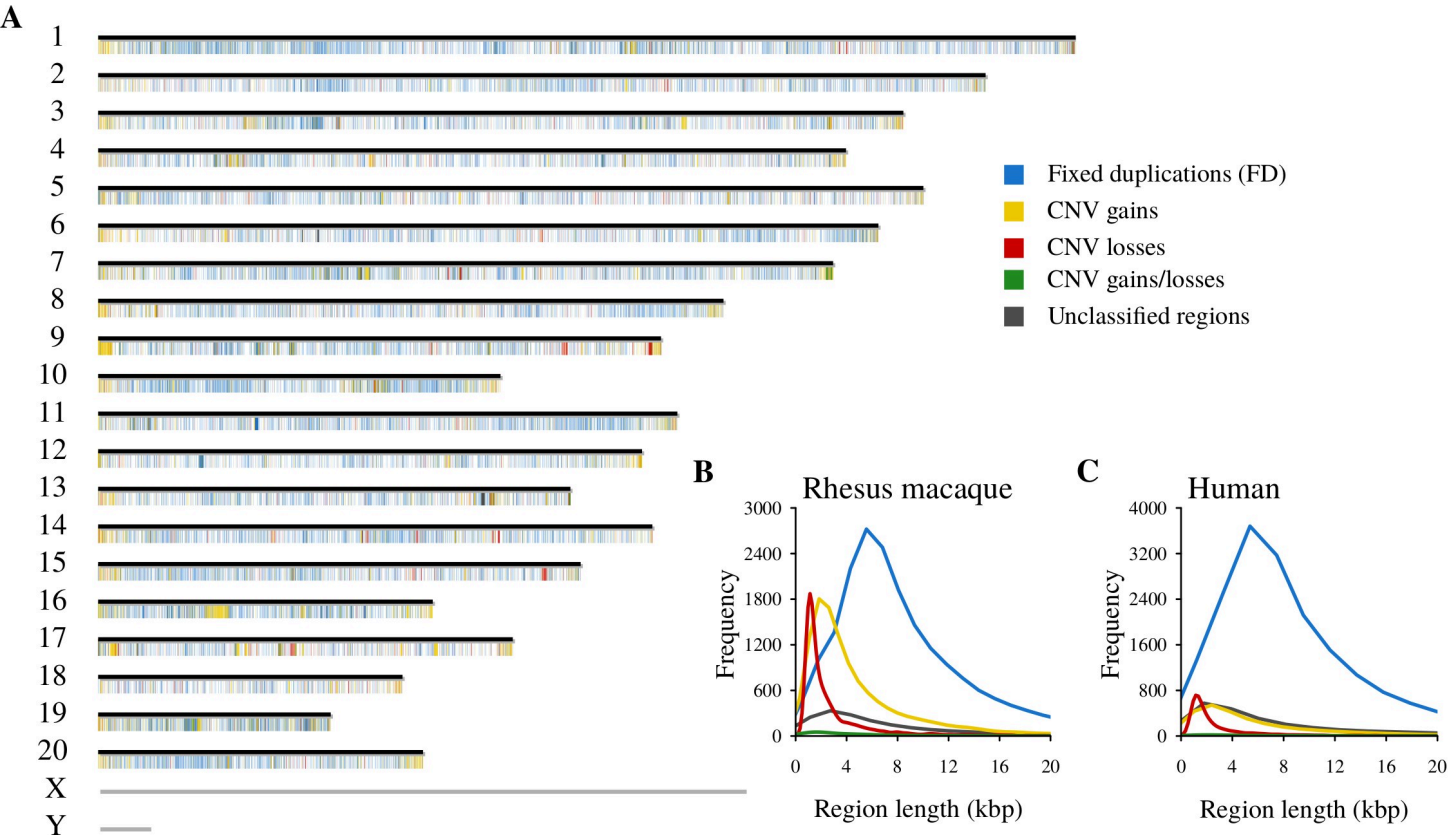
Landscape of the non-diploid regions in the rhesus macaque reference genome. (A) Genome-wide map of the non-diploid regions. Chromosomes are represented by horizontal bars. FD regions, CNV gains, CNV losses, CNV gains/losses, and unclassified regions are represented as color-coded marks. Mark width is proportional to the region length with a minimum mark width of 25 kbp for short regions for the sake of visualization. Sex chromosomes were not included in the analysis. (B) Length distribution of the non-diploid region types in the rhesus macaque genome. (C) Length distribution of the non-diploid region types in the human genome.

In rhesus macaques, non-diploid regions represent an important percentage of the genome (Fig 2A). FD regions cover 8.60% (of bps) of the complete build of the reference genome, while CNV gains cover 2.18%; CNV losses, 0.37%; CNV gains/losses, 0.07%; and unclassified non-diploid regions, 1.03%. Our copy number maps in humans show a higher prevalence of FD regions and unclassified regions compared to CNV regions (10.20% of FD regions, 1.60% of CNV gains, 0.18% of losses, 0.03% of gains/losses and 2.31% of unclassified regions). These results are reminiscent of observations in interspecies comparison for SNPs. That is, rhesus macaques have greater SNP diversity than do humans [31], which is consistent with the higher effective population size in macaques. However, the differences in the human and rhesus macaque sample size of our maps and in the quality of the two reference genomes can bias quantitative comparisons in different directions. Further work will be needed to determine the consistency of this observation in a more parallel comparative scenario (same sample selection criteria, sample size, similar assemblies, comparable genomic regions, etc.).

To validate our definitions of CNV and FD regions and our copy number calling, we compared our results with the aCGH results published by Lee et al. in 2008 [36]. After a liftOver conversion, we report 46% of Lee et al. [36] regions as non-diploid regions (28.6% of them as CNV regions). Overall, we recover a significant fraction of the regions detected by Lee et al. [36] despite the important differences between methods and sample sizes (see Methods for details).

FD regions appear to be the longest type of non-diploid regions (in terms of base pair length) in the rhesus macaque genome (Fig 2B). The same pattern holds in our human copy number maps (Fig 2C). Among CNV regions, CNV losses and gains/losses are shorter than CNV gains in both species (Fig 2B and 2C). The presence of big and old mosaic clusters around ancestral duplicons has been extensively reported in several species genomes [33,52–54]. We suspect that most of the length of these big clusters will appear as long FD regions, explaining the difference in length between FD and CNV regions. In addition, as stated above, CNV regions are more difficult to call reliably, resulting in more fragmented regions interrupted by unclassified segments that might be actually unresolved CNV segments (see Methods).

Non-diploid regions are known to be distributed widely along the human genome but to show regions of substantial clustering [33] and to be enriched in pericentromeric and subtelomeric regions [49,52,53]. Consistent with these previous observations, our human copy number maps show the expected clustering of human non-diploid regions in both pericentromeric and subtelomeric regions (S2 Fig). A genome-wide overview of FD regions, CNV regions, and unclassified non-diploid regions in the rhesus macaque genome shows a similar distribution (Fig 2A). Non-diploid regions are common (12.25% of bp) and widespread along the rhesus macaque reference genome with appreciable subtelomeric clustering of CNV gains (Fig 2A). Nevertheless, we cannot document pericentromeric clustering in the rhesus macaque genome due to the lack of resolution and annotation of centromeres in the genome assembly. Overall, the genome-wide distributions of CNV regions in rhesus macaque and human show similar features, suggesting that they are conditioned by the same mechanistic and/or selection constraints.

Interestingly, we observed some differences in the chromosomal density of non-diploid regions in the rhesus macaque genome (S3A Fig). Rhesus macaque chromosome 19 (which is orthologous to human chromosome 19) shows a particularly high density of all non-diploid classes of regions (FD regions, CNV gains, and unclassified non-diploid regions). This observation coincides with the genomic distribution of rhesus macaque segmental duplications [55] across chromosomes (S3B Fig). We independently retrieved information on mammal, primate, and rhesus-specific gene duplications [56] and explored their distribution across chromosomes (see Methods). There is a higher density of genes duplicated in mammals and primates on chromosome 19 compared to other chromosomes, but not of genes specifically duplicated in the macaque lineage (S3B Fig). Moreover, humans also show an especially high contribution of non-diploid regions (basically FD regions) on chromosome 19 (S4 Fig), which is known to have the highest gene density of all human chromosomes (more than double the genome-wide average) [57] and to have a high density of tandemly arranged families [57]. Rhesus macaque and human share a large proportion of genes duplicated in mammals and primates. These observations confirm that chromosome 19 has undergone particular segmental duplication activity during mammalian evolution with a corresponding genesis of new genes.

Additionally, in humans and other great apes, chromosome 9 is known to have large pericentromeric non-diploid regions [5]. Our human copy number map retrieves this feature showing a higher proportion of chromosome 9 length covered by non-diploid regions (S4 Fig). Interestingly, the rhesus macaque chromosome 15, orthologous to great ape chromosome 9, shows no particular enrichment in non-diploid regions (S3A Fig). Consequently, the large accumulation of non-diploid regions in the pericentromeric regions of human chromosome 9 probably happened in the last 26 million years, after the rhesus macaque lineage diverged from the hominoid lineage.

Many factors contribute to genome-wide patterns of duplications such as the core duplicon hypothesis proposed for primates [33] whereby duplications of diverse interspersed origin tend to cluster locally around core duplicons. Additionally, the intrinsic tendency of certain regions to accumulate higher levels of variability associated with specific structural, epigenomic, or mechanistic differences [56], [58] must also be taken into account. Finally, selective forces may have influenced the presence of spatially confined highly variable regions, their specific locations and/or the variable regions involved [59]. Analyzing the contributions of each of these forces in rhesus macaque FDs and CNVs, in particular for chromosome 19, is indeed of great interest and should be pursued in future work.

### Copy number profile of protein-coding genes

As a first step in exploring functionally relevant copy number changes between human and rhesus macaque, we identified those copy number alterations affecting protein-coding genes in each species. Protein-coding genes are among the best-resolved regions in every species because they are found in regions of lower complexity. RNA-seq data can enhance their detection and their higher conservation makes it possible to project their annotations from closely related species. In fact, rhesus macaque gene annotations take advantage of the projection of human annotations (genebuild documentation at Ensembl [60]). Despite this higher annotation quality, big differences are still expected between human and macaque. In particular, there exists an important delay in the projection of human annotations to macaque which therefore lacks some refinements introduced in recent years [61,62]. Consequently, comparisons based on species-specific genes must be considered with caution since completely equivalent information is available for only a subset of protein-coding genes.

Duplications encompassing all the exons of a specific transcript of a protein-coding gene imply a higher number of potentially functional gene copies. These additional gene copies can affect expression levels with additional downstream consequences. For this reason, we focused on the main isoforms of protein-coding genes in both species, and more specifically, on their exons (see Methods). We distinguished between three levels of gene copy number profiles according to the degree of overlap with non-diploid regions (Fig 1B, S1B and S5 Figs). First, we identified all genes with at least one of their exons showing non-diploid copy number (overlapping with FD, CNV, or unclassified regions). Second, among the genes that overlap with FD or CNV regions, we distinguish between genes that are just partially overlapping them (*partial FD genes* and *partial CNV genes*, respectively) and genes having 90% or more of their protein-coding sequence duplicated and/or varying in copy number (*genes within FD regions* and *genes within CNV regions*). Third, among the genes within FD or CNV regions, we identified those with a constant copy number within each sample across all exons along the whole protein-coding region (*complete FD genes* and *complete CNV genes*). These two last groups of genes are protein-coding genes that have more than one or a variable number of presumably functional copies within our samples (see Methods). The genes within FD or CNV regions without a constant copy number along the coding regions per sample are termed *composite FD genes* and *composite CNV genes*. The membership of a given gene in one of the aforementioned categories will henceforth be referred to as the *copy number profile* of that gene.

**Fig 3:**
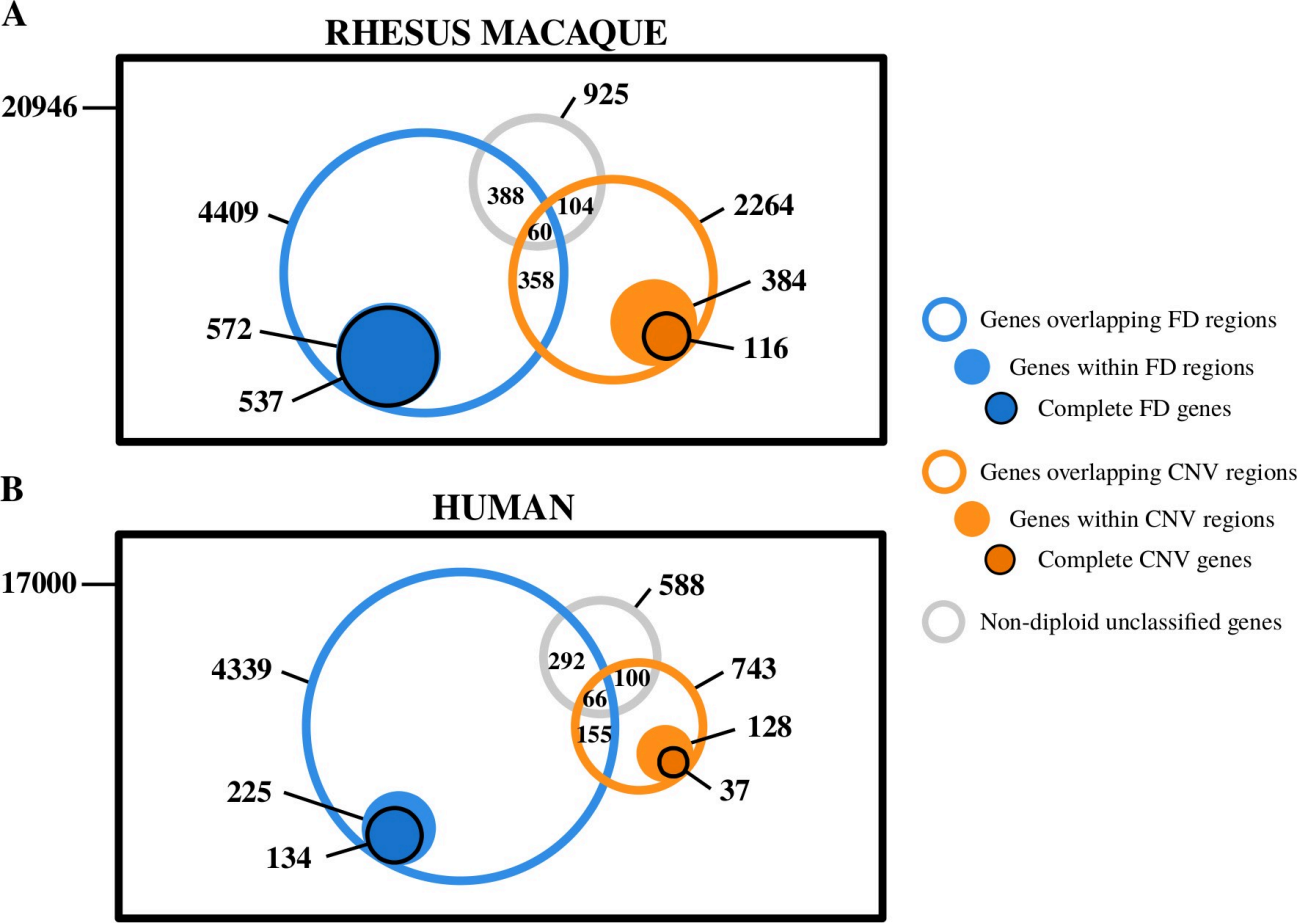
Copy number profile of rhesus macaque protein-coding genes. (A) Venn diagram representing, first, the entire set of rhesus macaque protein-coding genes considered (black square; a total of 20,946 protein-coding genes, 6,728 of which overlap with a non-diploid region); second, the number of genes overlapping (at least 1bp) with FD regions (blue), CNV regions (orange) and unclassified regions (grey) in open circles; third, genes within FD regions (blue) and CNV regions (orange) light-colored circles; and fourth, complete FD genes (blue) and complete CNV genes (orange) in dark-colored circles. All areas are approximations. (B) Equivalent Venn diagram corresponding to the human protein-coding genes.

Using these categories, we classified every protein-coding gene in the rhesus macaque and human genomes (Fig 3A and 3B, respectively). Our maps show that a large proportion (32.12%, 6,728 genes) of the rhesus macaque protein-coding genes overlap with either FD regions (21.05%, 4,409 genes), CNV regions (10.81%, 2,264 genes) or unclassified non-diploid regions (4.42%, 925 genes). We also observed that gene length is inversely correlated with the proportion of the protein-coding gene sequence having a non-diploid copy number (S6A and S6B Fig). This suggests that these overlaps are driven by the size of genes and non-diploid regions and that most of them may be neutral. Nevertheless, in the rhesus macaque genome, there is a significant proportion of protein-coding genes completely located within non-diploid regions (S6C Fig). These genes tend to be small (S6D Fig) because they are more easily encompassed completely by a duplication.

As shown in Fig 3A, in rhesus macaque, 93.88% (537) of the 572 protein-coding genes within FD regions are complete FD genes, a percentage that contrasts with the 30.21% (116) of the genes within CNV regions being complete CNV genes. This striking difference may be partially due to the increased uncertainty in allelic calls in CNV regions compared to FD regions. The presence of more than one allele increases the variability of confidence intervals and complicates precise allele calling. Thus, the figure of 30.21% is likely to be an underestimate.

### Comparing copy number profiles between rhesus macaque and human protein-coding genes

**Fig 4:**
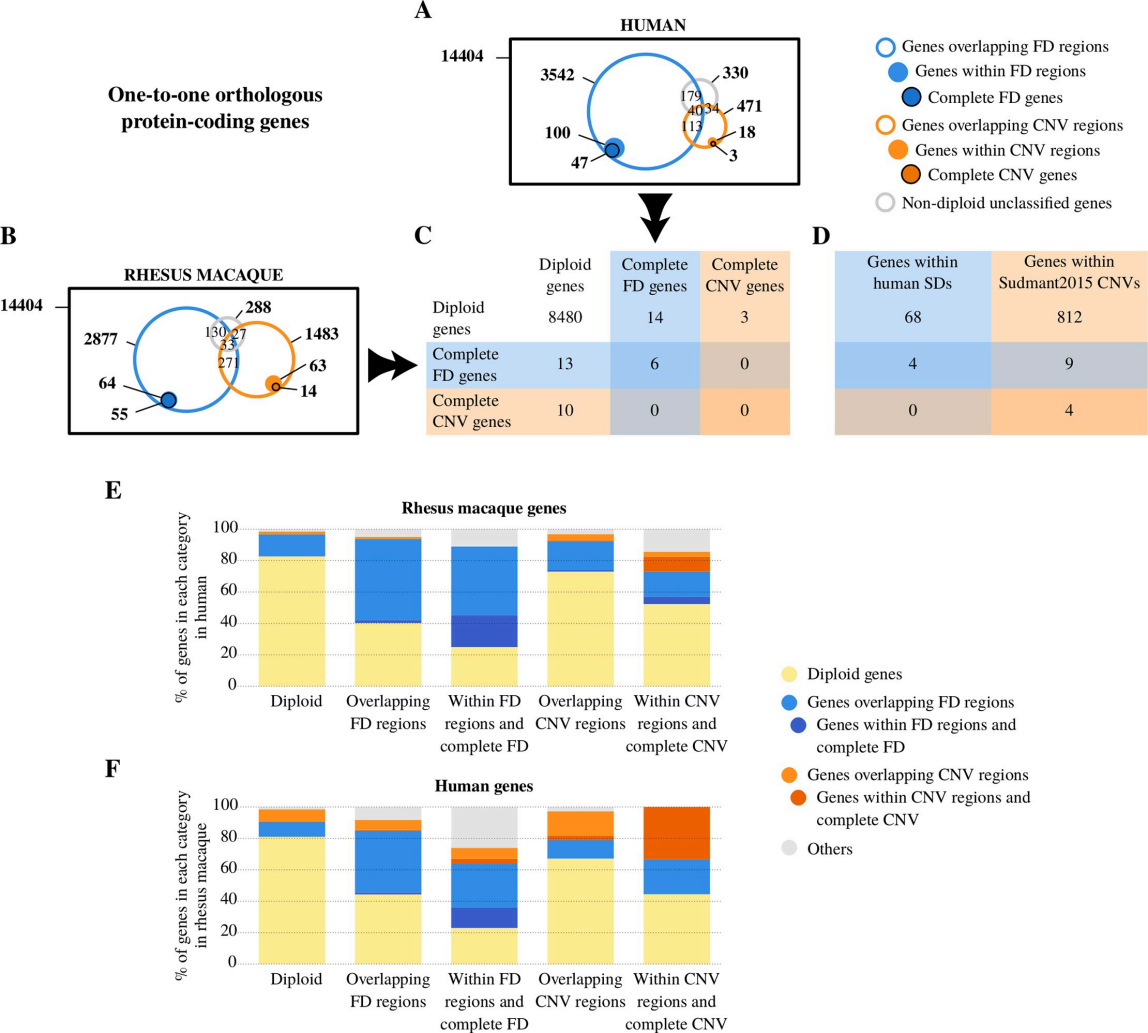
Copy number profile of one-to-one orthologous protein-coding genes in rhesus macaque and human. (A) and (B) Venn diagrams representing, first, the entire set of one-to-one orthologous protein-coding genes in each species; second, the number of genes overlapping with FD regions (blue), CNV regions (orange) and unclassified regions (grey) in open circles; third, genes within FD regions in (blue) and CNV regions (orange) light-colored circles; and fourth, complete FD genes (blue) and complete CNV genes (orange) in dark-colored circles. All areas are approximations. (C) Colored table representing the overlap in diploid, complete FD genes, and complete CNV genes of one-to-one orthologous genes from each species (see S9A Fig for the complete version of this table). (D) Colored table representing the number of human genes with one-to-one orthologs in rhesus macaque intersecting with human segmental duplications and Sudmant et al. [63] human CNV calls. Only diploid, complete FD, and complete CNV macaque genes were considered in this summarized version of the table (see S9B Fig for the complete version of this table). (E) and (F) Conservation of copy number profiles of one-to-one orthologous genes between rhesus macaque and human. Each column represents a copy number profile in each species. The Y-axis represents the percentage of genes in each column with a given copy number profile in the other species. The second (fourth) column in each plot, corresponding to genes overlapping FD (CNV) regions, includes genes within FD (CNV) regions and complete FD (CNV) genes, shown in the third (fifth) column in each plot. “Others” category includes genes overlapping with unclassified non-diploid regions and genes overlapping with both FD and CNV regions.

Our catalog of genic segments that are non-diploid allows us to identify differences in copy number between the two species that are likely to have functional consequences. As explained above, the quality of the gene annotations in both species is different. To address this situation, we compared the copy number profile of human and rhesus macaque orthologous gene pairs (see Methods; S3 Table). In this way, we focus on changes in copy number variability in genes that can be compared between species according to the best available annotations (although we may be missing other interesting cases due to uncertainty in annotations). We compared human-rhesus macaque orthologous pairs from the 14,404 one-to-one orthologies (genebuild documentation at Ensembl [60]) where much less functional divergence is expected (Fig 4). In addition, we also performed the same analysis with all pairs of orthologs with consistent results but a more complicated interpretation (S7 Fig). Private human genes and private rhesus macaque genes are not considered in this analysis because they lack orthologs in the other species [62]. Here, it is worth recalling that this comparison aims to detect those genes in the rhesus genome showing copy number variation profiles that differ from the human profile and therefore may not be suitable as models for human gene/phenotype relationships.

Comparison of copy number variability in human and macaque one-to-one orthologous genes shows that both CNV maps are highly comparable with most of the orthologous pairs being diploid in both species (Fig 4A and 4B). Despite this global coherence, both maps still show some interesting differences. In particular, while macaque genes are less commonly overlapping FD regions (2,877 and 3,542 genes, respectively), they are more often overlapping CNV regions than human genes (1,483 and 471 genes, respectively). These differences can be a consequence of a higher copy number variability in the rhesus macaque genome, but a bias due to differences in sample number and selection criteria cannot be disregarded.

Interestingly, we found genes annotated as one-to-one orthologs that are complete FD genes either in rhesus macaque or in human (55 and 47 genes, respectively; Fig 4A and 4B). These results point towards unresolved duplications in the human and rhesus macaque assemblies involving genes that might be contributing to basic phenotypic differences between human and rhesus. In a direct comparison between one-to-one orthologous genes (Fig 4C), we found 6 shared complete FD genes between the two species. We observed that 13 one-to-one orthologous pairs are complete FD genes in macaque and diploid in humans, while 14 one-to-one orthologous pairs are complete FD genes in humans and diploid in rhesus macaque. In all these cases, the incompleteness of the reference genomes has led to their misidentification as one-to-one orthologs. Regarding complete CNV genes, we detected 10 cases of one-to-one orthologs that are complete CNV genes in rhesus macaque and diploid in human and three one-to-one orthologs complete CNV genes in human that are diploid in macaque. Finally, when considering the complete set of orthologies (not only one-to-one orthologs), we found nine cases of shared complete CNV genes (S7C Fig), pointing to genes that might be intrinsically prone to be copy number variable.

Despite the expected high conservation of diploidy of genes (more than 80% of diploid genes in one species are also diploid in the other species), diploid genes in one species can present all types of copy number profiles in the other species (Fig 4E and 4F). Moreover, genes related to FD regions tend to conserve their relationship with FD regions in the other species more than CNV related genes do. So overall, diploid status is the most conserved state between human and macaque orthologs, which is expected, followed by FDs and finally CNVs, which is the least conserved copy number profile (Fig 4E and 4F).

Given the extremely high number of pairs of orthologous genes that are diploid in both species, we statistically evaluated the maintenance of non-diploid copy number profiles between human and macaque one-to-one orthologs. To this aim, we simulated (10,000 times) a random distribution by shuffling copy number categories against genes after excluding all diploid-diploid pairs. We tested the significance of the observed value of maintenance of non-diploidy (either overlapping FD, within FD, overlapping CNV or within CNV) against the obtained distribution. Our results show that there are more changes from diploid to non-diploid behavior between human and macaque orthologs than expected by chance (p-value < 0.0001). Interestingly, these results show that, despite genome-wide similarities in copy number distributions and the general conservation of diploid genes, FD and CNV profiles in genes are usually not shared between human and macaque. This suggests that structural variation is an important contribution to the genetic and phenotypic divergence between human and macaque and reinforces our interest in the possible implications of these differences.

Although we have observed some discrepancies between our human and rhesus macaque maps of structural variation, our results show that the maps are highly comparable at the gene level. For the sake of completeness, we also considered the annotation of segmental duplications in the human genome and the human CNV dataset from Sudmant et al. [63]. Annotated segmental duplications include duplications more divergent than the ones targeted by our approach. Consequently, most of the genes within human segmental duplications with one-to-one orthologs in rhesus macaque appear to be diploid in the latter (68 genes). We retrieved four complete FD genes in macaque that have one-to-one orthologs within human segmental duplications (Fig 4D).

The Sudmant et al. [63] CNV dataset used a different methodology, sample size and selection criteria of individuals than our human panel. Sudmant et al. [63] included a larger number of human genomes and was intended to detect intra-population copy number variability. In contrast, our panel of human samples was intended to be compared with the rhesus macaque genome and was designed to detect inter-population copy number variability. In this way, Sudmant et al. provide a more comprehensive dataset that helps to discriminate completely fixed regions from those with low-frequency CNV alleles in humans. Therefore, it is not surprising that most of the genes within the Sudmant et al. [63] dataset with one-to-one orthologs in rhesus macaque are diploid in rhesus macaques (Fig 4D). We also identified nine complete FD genes *(APOL2*, *ZNF418*, *PPP2R5C*, *ZNF33B*, *MT1E*, *TXN*, *TMEM211*, *MT1G*, and *ZNF92*) and four complete CNV genes (*CCDC115*, *IMP4*, *KRTAP1-5*, and *KRTAP1-1*) in the rhesus macaque genome that have one-to-one orthologs within the Sudmant et al. [63] CNV dataset.

### Disease associations for genes with different copy number between humans and rhesus macaques

**Fig 5:**
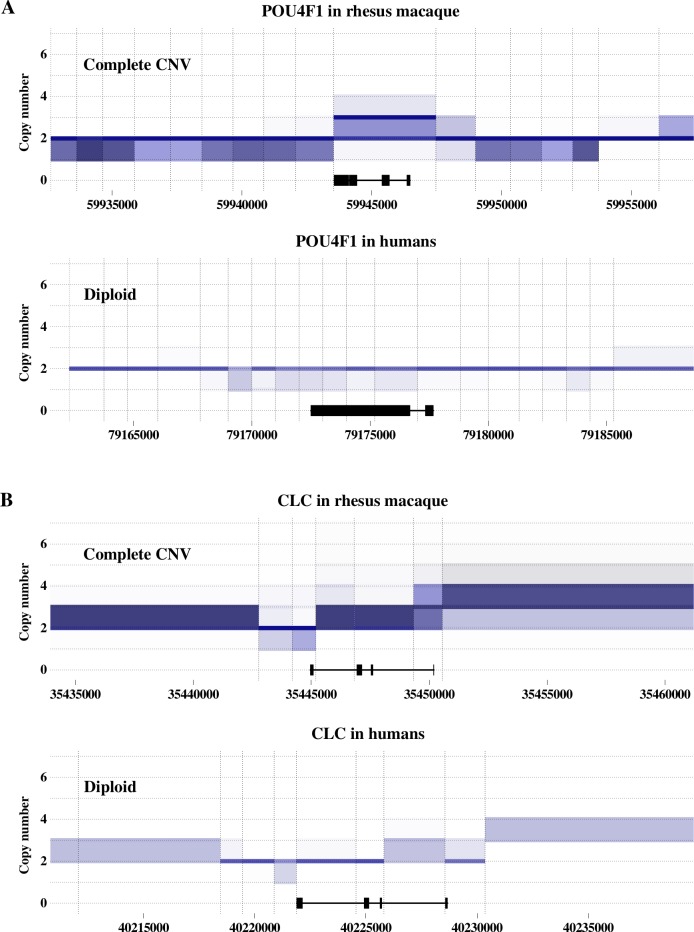
**Copy number along the sequence of *POU4F1* (A) and *CLC* (B) in rhesus macaques (top) and in humans (bottom).** The X-axis represents the genomic sequence and the Y-axis represents the distribution of copy numbers across all our samples in each window. Vertical dashed lines represent window limits, and the horizontal black line at the bottom represents the corresponding gene length and black boxes represent exons. Copy-number for each individual can be either a single integer (e.g., 2) or an interval spanning several integers (e.g., 2–3; see Methods). Single-integer copy numbers are represented by a thick line along the length of the window, whereas, interval copy numbers are shown as a box spanning the whole of the interval, including the flanking integers. Color shade gradients are proportional to the number of individuals with the corresponding copy number interval or integer, with darker shades meaning more individuals. (A) *POU4F1* is called as a complete CNV in rhesus macaque since, in all the windows where the gene exons are located, there are individuals with copy number 2 (diploid), individuals with copy number interval 2–3, individuals with copy number 3, and individuals with copy number interval 3–4 (see allele calling procedure in Methods). Differently, *POU4F1* is called as diploid in humans since all the windows encompassing its exons have individuals with copy number 2 and individuals with copy number interval 1–2. Ensembl ID numbers for *POU4F1* are ENSMMUG00000030683 and ENSG00000152192 for rhesus macaque and human, respectively. (B) *CLC* is called as a complete CNV in rhesus macaque since all the windows that cover its exons contain individuals with non-overlapping copy number intervals (1–2, 2, 2–3, 3–4, 3–5, etc.), and as diploid in humans since all windows covering its exons have individuals with copy number 2 and with intervals including copy number 2. Ensembl ID numbers for *CLC* are ENSMMUG00000009527 and ENSG00000105205 for rhesus macaque and human, respectively.

Copy number variation is one of the main sources of phenotypic variability between individuals in a population. Consequently, differences in the copy number profile between rhesus macaque and human orthologous genes have the potential of leading to interspecies differences in specific phenotypes. In particular, we studied the relevance for human disease of the few one-to-one gene pairs that have a different copy number in the two species. We explored complex disease-related genes by intersecting our genes of interest with the GWAS catalog database [64] and the DisGeNET database [65]. Table 1 shows the list of the 18 identified associations of human disease and genes having relevant differences in their copy number profile between human and macaque. Fig 5 shows the copy number of all individuals in both species along with the sequence of two chosen interesting examples (all cases in Table 1 can be found in S10–S25 Figs). Interestingly, several important complex disease-related genes have relevant differences in copy number profile between human and rhesus macaque (Table 1), with some of them associated with diseases for which rhesus macaque is a model organism, such as Alzheimer's disease, multiple system atrophy, schizophrenia, autism, amyotrophic lateral sclerosis, chronic kidney disease, and asthma [20–29]. In particular, we found several genes related to complex neuropsychiatric and neurodegenerative disorders differ in copy number between rhesus and humans.

**Table 1:** 

Human Gene	Disease	Database	Human Copy number Profile	Rhesus Copy number Profile
*TXN*	Late-onset Alzheimer’s disease	GWAS Catalog	Diploid & within Sudmant2015 CNVs	Duplicated and fixed as a whole
*ENY2*	Multiple system atrophy	GWAS Catalog	Diploid	Duplicated and fixed as a whole
*MPC2*	Schizophrenia	GWAS Catalog	Diploid	Duplicated and fixed as a whole
*PPP2R5C*	Autism	GWAS Catalog	Diploid & within Sudmant2015 CNVs	Duplicated and fixed as a whole
*ILF2*	Multiple myeloma	GWAS Catalog	Diploid	Duplicated and fixed as a whole
*SYCP3*	Azoospermia Arrest of spermatogenesis	DisGeNET	Diploid	Duplicated and fixed as a whole
*POU4F1*	Alzheimer’s disease	GWAS Catalog	Diploid	CNV as a whole
*CLC*	Schizophrenia	GWAS Catalog	Diploid	CNV as a whole
*VAPB*	Amyotrophic lateral sclerosis Spinal muscular atrophy	DisGeNET	Diploid	CNV as a whole
*CCDC115*	Congenital disorder of glycosylation	DisGeNET	Diploid & within Sudmant2015 CNVs	CNV as a whole
*APOL2*	Chronic kidney disease	GWAS Catalog	Duplicated and fixed as a whole & within Sudmant2015 CNVs	Duplicated and fixed as a whole
*HIST1H2AC*	Narcolepsy with cataplexy Lung carcinoma Schizophrenia	GWAS Catalog	Duplicated and fixed as a whole	Diploid
*NAT8*	Chronic kidney disease	GWAS Catalog	Duplicated and fixed as a whole	Diploid
*JUND*	Asthma	GWAS Catalog	Duplicated and fixed as a whole	Diploid
*HSD17B7*	Dupuytren contracture	GWAS Catalog	Duplicated and fixed as a whole	Diploid
*SPDYE4*	Attention deficit hyperactivity disorder	GWAS Catalog	Duplicated and fixed as a whole	Diploid
*PVRIG*	Acute coronary syndrome	GWAS Catalog	Duplicated and fixed as a whole	Diploid
*HSPB8*	*Trypanosoma cruzi* seropositivity Charcot-Marie-Tooth disease Distal hereditary motor neuropathies	GWAS Catalog	Duplicated and fixed as a whole	Diploid

Given these associations, we wondered if non-diploid genes and/or human-macaque orthologs with different copy number profiles were more frequently associated with complex diseases than expected by chance. To this aim, we performed simulations following the same strategy previously used for testing conservation of non-diploidy and compared the copy number profile of genes in each one of the species with the genes appearing in the GWAS catalog and in DisGeNET (with a score > = 0.06, which only includes associations reported by curated sources). We found that both human (p-value < 0.0001 for the GWAS catalog and p-value = 0.0981 for DisGeNET) and rhesus macaque (p-value = 0.0004 for the GWAS catalog and p-value = 0.0082 for DisGeNET) non-diploid genes are more related to complex diseases than expected by chance. We also found that genes with different copy number profiles between human and rhesus macaque are more associated with complex diseases than expected by chance (p-value = 0.0004 for the GWAS catalog and p-value = 0.0082 for DisGeNET). Moreover, diploid genes in one species that are non-diploid in the other one are also enriched in the GWAS catalog (p-value < 0.0001), but their enrichment is not significant in DisGeNET (p-value = 0.0882). Overall, these results reinforce the relevance of non-diploid genes in complex diseases and the importance of paying special attention to genes showing copy number differences between species when working with complex diseases in model organisms.

Among the genes affected by these differences, we found that *PPP2R5C* is diploid in humans and complete FD in rhesus macaque. This gene encodes a regulatory subunit of the protein phosphatase-2A (*PP2A*), an intracellular serine/threonine phosphatase, which is a tumor suppressor protein. Our analysis shows that *PPP2R5C* probably has more functional copies in the rhesus macaque genome than in the human genome (S17 Fig). This observation concerning *PPP2R5C* is of special relevance because this gene has been related to autism [66], a disorder for which rhesus macaques are increasingly used as a model organism [23]. Consequently, caution should be necessary when interpreting experiments targeting aspects of autism that could be affected by this gene.

*POU4F1/BRN3A* is a homeobox 1 neuronal transcription factor involved in axonal projection and related to cell differentiation and survival [67] (Fig 5A). *POU4F1/BRN3A* has been associated with Alzheimer’s disease [68]. It interacts with *HIPK2*, a serine/threonine kinase that has been shown to be involved in an amyloid-based pathology leading to the survival of dysfunctional neurons [69]. Moreover, this interaction regulates the survival of sensory neurons [70], suggesting a direct connection to Alzheimer’s disease. This gene shows a higher copy number in most macaques compared to humans which might affect its role in Alzheimer’s disease. Importantly, in this case, it is possible to find macaque individuals diploid for this gene, suggesting that these individuals are likely to be more suitable models for this disease (especially for studies focused on this gene). Interestingly, alteration in the copy number of a region close to *POU4F1/BRN3A* is also associated with autism spectrum disorder [71].

Another macaque complete FD gene that is diploid in humans is *SYCP3* (S11 Fig), a major structural component of the synaptonemal complex. Heterozygous mutations in this gene have been associated with male infertility [72]. This mutation truncates the protein and reduces the ability to interact with the wild type protein probably affecting *SYCP3* fibre formation, which can engage in extensive interactions with DNA [73]. As this mutation has been suggested to compromise spermatogenesis via a dominant negative interference, the additional copy in rhesus macaques may make them more susceptible to this disorder (although it might result in a milder condition). This example suggests that differences in copy number may lead to different effects depending on the mechanism of action of the corresponding protein and the consequences of the corresponding mutations.

The situation is even more complex in the case of *MPC2* (S18 Fig), *CLC* (Fig 5B), and *HIST1H2AC* (S24 Fig), which are all associated with schizophrenia. These genes have differences in copy number between human and macaque and show different types of changes in copy number profile (see Table 1). None of these associations has been functionally validated, making the effect on schizophrenia of differences in copy number uncertain. Our data suggest that usage of rhesus macaque as a model to study the effects of these genes should proceed with caution. As a whole, our results show that the actual number of copies of a specific gene in an individual’s genome should be considered when using rhesus macaques to model a disease process.

## Discussion

The rhesus macaque is one of the most ecologically successful primate species [74] and the nonhuman primate most intensively used as a model for human disease [20]. However, the genomic structural variation of this species has received little attention. Here, we report and characterize the first rhesus macaque high-quality genome-wide landscape of CNV regions and FD regions, employing data from 198 individuals. We used a consolidated approach based on the depth of read coverage for each region. This method is suitable for estimating genome-wide copy number in species that have draft quality genome assemblies (see Methods). We were able to confidently define CNV gains, gains/losses and losses, as well as FDs in the population. Acknowledging the existence of non-diploid regions of uncertain status (labeled as unclassified) leads to a more reliable map but also calls for caution in the interpretation of genome-wide copy number patterns.

Differences in copy number between closely related species are an invaluable source of information to understand and identify the molecular bases of species-specific traits. To define potentially relevant differences in the copy number profiles of human and macaque genes at a population level, we used the same methodology to generate a human copy number map based on a subset of highly diverse samples selected to maximize the detection of inter-population variability. In agreement with previous reports in humans [33,49,52,53], we observed non-diploid regions clustered in certain regions of the rhesus macaque genome, including subtelomeric regions. Our genomic landscape shows an enrichment of non-diploid regions in rhesus chromosome 19 and also found enrichment in the orthologous human chromosome 19. Gene-based phylostratigraphy [56] concerning duplicated genes in rhesus macaque chromosome 19 shows an enrichment of genes duplicated across mammals and in primates, but not macaque-specific genes, suggesting that the accumulation of duplicated genes in chromosome 19 occurred before the macaques diverged from humans and other hominoids. In contrast, the known and described accumulation of non-diploid regions in great ape chromosome 9 [5] does not appear in its rhesus macaque ortholog, chromosome 15. This suggests a more recent hominoid lineage-specific origin for this enrichment.

Despite the differences in the human and macaque sample sets, genome assembly qualities, and population structures, we obtained comparable numbers for non-diploid regions in our macaque and human maps. However, FDs were more often observed in the human map, which could be related to the better quality of the human genome assembly. In contrast, the higher numbers of rhesus macaque CNV gains, gains/losses, and losses could point to the higher genetic diversity in macaques, although additional work will be needed to clarify the potential effects of sampling design and population structure on these observations.

In order to investigate the possible impact of structural variants in macaques and how it differs from that in humans, we focused on protein-coding genes. Assessing the functional impact of structural variants affecting genes is not straightforward. Therefore, we developed a strict protocol that identifies CNV regions or FD regions affecting complete genes. We reasoned that changes in the number of copies of entire genes would more likely be associated with specific phenotypic changes than partial duplications.

In this way, we identified 2,264 macaque genes (743 human genes) affected by CNV regions, 116 of them being completely gained, gained/lost or lost in rhesus macaques (37 in humans). Consistent with the genome-wide CNV distributions, these numbers are larger in macaques than in humans. However, this difference in global numbers can be, at least partially, attributed to the different criteria used for sample selection in both maps and the differences in the quality of gene annotation between these two species since recent refinements of human annotations [62] remain to be projected to rhesus. Focusing on one-to-one gene orthologs between the two species allows for a more defensible comparison yet more limited in number.

About one-third of the human orthologs of our macaque complete CNV and FD genes could be detected with an equivalent classification in at least one of the analyzed human datasets. Due to the extensive characterization of the human genome, the rest of the complete non-diploid proteins in rhesus macaque (10 complete CNV genes and 13 complete FD genes) are highly likely to exhibit a true species difference. In fact, we found that, after controlling for trivial conservation of diploid states, changes from diploid to non-diploid states between human and macaque orthologs are more common than expected by chance. This excess of changes in copy number suggests a relevant role of copy number variants in the divergence of genic repertoires between both species. Interestingly, we found that at least 18 of the genes with different copy number levels between human and macaque are associated with various diseases in human [64,65], although none of them showed human inter-population copy number variability. Among these diseases, we found several neuropsychological diseases, such as Alzheimer’s disease, autism spectrum disease, ADHD and schizophrenia, as well as male infertility and chronic kidney disease. Moreover, we found that, for both human and macaque, non-diploid genes are enriched in human complex-disease gene datasets. Similarly, changes in copy number profile between human and macaque are also significantly enriched in complex-disease genes.

Different changes in copy number profile represent different challenges for using macaque as a model organism to study a given disease. For instance, CNV genes in macaque whose orthologs are fixed (diploid or duplicated) might allow for the selection of individuals with human-like copy numbers. This would be the case of genes such as *POU4F1* for Alzheimer's disease or *CLC* for schizophrenia. In the opposite situation, with human CNV genes and fixed macaque orthologs, the copy number is unlikely to be essential for suffering the disease, although it might affect disease susceptibility. We only found this situation for some genes showing intra-population copy number variability in humans, such as *PPP2R5C* for autism or *APOL2* for chronic kidney disease. In these cases, macaque results could be more reliably extrapolated to subpopulations of human individuals showing the same copy number. When both orthologs are variable in copy number, such as *CCDC115*, associated with congenital disorder of glycosylation, careful selection of macaque individuals could potentially permit the representation of specific human populations. Finally, when both species show a fixed and different copy number, no macaque individual mimics the human configuration, signifying an important and intrinsic limitation of rhesus macaque as a model organism for the study of these genes. This situation holds for genes such as *MPC2*, associated with schizophrenia, *SYCP3*, with azoospermia, and *NAT8*, with chronic kidney disease.

It is clear that the actual effect of these differences in copy number can be variable. In many cases, it could be inconsequential, especially in normal conditions, but we note that disease conditions imply an imbalance such that the presence of additional or fewer functional copies might affect disease risk. Although this means that the relevance of these effects is difficult to predict, we propose that it is worth controlling for these potential confounding factors when possible. The list of disease-associated genes with different copy number profiles presented here is necessarily incomplete and further research remains to be done to define the actual impact of each of these genes and others with differences in copy number. Consequently, we believe that our dataset of genes with different copy number profiles between human and rhesus macaque represents a valuable resource for those researchers using rhesus macaque as a model organism.

The genomic differences between human and rhesus macaque that we report here may be relevant to the use of rhesus macaques as a model system, in particular, for those diseases that we have identified as being related to genes with structural variant differences. One possible strategy for determining the significance of these observed differences would be to compare levels of mRNA or protein expression for the genes that show copy number differences. Expression levels of different allelic variants in normal and disease conditions could be considered in order to detect any effect of different copy numbers of these alleles. Not less important is the potential for these changes in copy number to explain observed interspecies differences in disease susceptibility, progression, prognosis or treatment. Nowadays, the power of defining individual genetic profiles to ensure the best possible personalized treatment is commonly recognized. However, the importance of considering these differences in research with model organisms is rarely acknowledged. Our work defines differences in gene copy numbers that should be considered when designing disease-related experiments in a model organism or translating the obtained results to humans.

## Methods

### Sampling and data generation

We use sequence data from an initial panel of 315 rhesus macaques. This set includes 113 previously published samples [31] and 202 newly sequenced samples. WGS was performed using the Illumina HiSeq 2000 platform, generating 100-bp paired-end reads. The final dataset contains 198 individual rhesus macaques from eight research colonies (S1 Table).

For comparison, with humans we used an initial panel of WGS data from 50 human samples specially selected from the Simons Genome Diversity Project [51] to cover a great part of human diversity.

### Copy number calling

In order to call copy number genome-wide with high confidence, we used a *Hidden Markov Model* (HMM) algorithm [75] for copy number estimation as described in detail in Serres-Armero et al. [43]. This approach applies the HMM on top of continuous copy number inferences based on read depth [48].

We here describe the procedure adapted to the data in this study:

1. Repeat- and over-represented k-mer- masking of the assembly. We started masking all of the interspersed and simple repeats annotated in the assembly Mmul 8.0.1/rheMac8 (GRCh37/hg19 for humans) with RepeatMasker [76] and Tandem Repeat Finder [77]. Then, in order to mask over-represented k-mers, we divided the assembly into 78 bp sliding k-mers. These k-mers were mapped to the assembly using GEM mapper [78]. All positions with more than 20 k-mer placements were masked. Masked assembly is available upon request.
2. Mapping of WGS reads to the masked assembly. WGS reads were mapped to the previously masked version of rheMac8 using GEM mapper [78]. In order to prevent underestimation of the copy number in the surroundings of the masked repetitive regions, we masked the 78 bps flanking all masked regions after the mapping.
3. Defining genome windows. We divided the assembly in 1 kbp non-overlapping windows of non-masked sequence and we calculated the read depth in these windows using mrCaNaVaR [48].
4. Raw copy number estimation per window. Control diploid regions were defined by mrCaNaVaR as those kept after removing regions with outlier read depths until a Poisson distribution is acquired. Mean read depth per window for the set of control diploid regions was taken as the read depth corresponding to the diploid copy number (copy number 2). This read depth was used as corresponding to copy number 2 to scale the mean read depth in all the other windows to a raw copy number (continuous value directly proportional to the mean read depth). This procedure takes into account GC content and corrects for it with mrCaNaVaR.
5. Integer copy number probability estimation with HMM [75]. We used an HMM to estimate the probability of a given window having an integer copy number from its raw copy number. In this HMM, the observed values are the raw copy number estimates per window, the hidden states are real integer copy numbers (0, 1, 2, 3,…, 20), and the transitions between states are changes in copy number between adjacent windows. The HMM hidden states for high copy numbers were set as intervals (21–100, 101–500, and 501–1000) instead of integers due to the correlation between copy number and noise. Emission probabilities were extracted from the corresponding read-depth distributions. For low copy number states, these distributions were defined as normal distributions with mean equal to the corresponding copy number (μ_N_ = N) and standard deviation $\sigma_{N}=\sigma_{CR}\sqrt{N/2}$, where *σ_CR_* is the standard deviation in control regions. For copy numbers above 20, emission distributions were a mixture of the corresponding normal distributions weighted proportionally to the estimated frequencies of each copy number. We used the Baum-Welch algorithm [79] to train the transition matrix of this HMM until convergence with a random set of samples that were excluded from further analysis. Finally, in order to predict the probability of each of the hidden states for each window in each individual, we used the forward-reverse algorithm coded in the Pomegranate Python package.
6. Local population-based re-genotyping. We applied a population-based correction for noisy local copy number estimates. We used Bayes’ theorem [80] to estimate the probability of observing a copy number in a given window for a given individual given the distributions corresponding to the different copy number states. In this correction, the prior probability of a given copy number in a given window (*p(N)*) was the only parameter that changed locally. The prior probability was set as the average probability of this copy number across all individuals in five consecutive windows centered at the window of interest. See Serres-Armero et al. [43] for further information.
7. Defining copy number intervals with a confidence level of 99%. For each window and each individual, we ranked all the possible copy number states by their probability. Then, starting with the top copy number state, we increased the copy number interval assigned to that particular window, for example, from (3) to (3–4) to (3–5), until the cumulative probability reached 99%. The underlying actual copy number will belong to this interval with 99% confidence.

### Sample quality control

Many factors can affect the coverage of a given sample apart from its copy number and GC content. Cell immortalization, library preparation, sequencing biases, and divergence to the reference, among other factors, can alter copy number inferences from the depth of coverage [6,81]. Since our method is very sensitive to the width of copy number intervals, we needed to control for external (i.e., experimental) factors affecting variance in coverage, noise, sequencing artifacts, outliers, hidden population substructure, among other factors. To avoid confounding factors in our copy number estimations as much as possible, we performed a very strict sample quality control, which is described below.

Our starting dataset includes 387 samples from three different batches: 113 high-coverage published samples (batch 1) with a mean raw coverage of ~35x; 74 low coverage published samples (batch 2) with a mean raw coverage of ~8x; and 202 high-coverage new samples (batch 3) with a mean raw coverage of ~32x. We excluded samples according to the following criteria:

1. Quality control filters of the pipeline removed 66 samples. These filters detect systematic deviations of the expected behavior of genome-wide read coverage distribution affecting the assumptions of our CNV calling approach:
  - Standard deviation of the coverage in diploid control regions: excluded samples higher than 0.5 (S26A and S26B Fig).
  - Kolmogorov-Smirnov statistic [82] for normality of the distribution of coverage: excluded samples lower than 0.03 (S26A Fig).
  - Pearson correlation coefficient [83] between neighboring windows: excluded samples greater than 0.15 (S26B Fig).
2. 5 samples were used for HMM training and hence, removed from the final dataset.
3. We detected a batch effect leading to a systematic increase of non-diploid calls in batch 2 (S26C Fig). As this batch was comprised of low-coverage samples (~8x) and our approach relies on read depth, a lower accuracy was expected for this set [48]. However, given this systematic effect, we removed the whole batch.
4. PCA-based detection of suspicious outliers. We excluded the samples segregating from all the main cluster in two consecutive rounds of copy number PCA according to visual inspections:
  - The first PCA revealed a subgroup of outliers with particularly low mean genome-wide coverages (S26D Fig). Given the dependence of our approach with read depth, we established a threshold based on these outliers of 11.7x raw coverage. So, we removed these outliers and a few additional samples that, despite not showing genome-wide divergences, were considered less reliable. This filter removed 16 samples.
  - The second PCA separates a major cluster from a subset of samples with a dispersed distribution. Preliminary analyses, including this subset, showed that most FDs and CNVs were private to these samples, driving a large part of our results. The segregation of this subset was not associated with the origin of the samples (S26E Fig), even though a SNP-based PCA clearly distinguished between individuals of Chinese and Indian origin (S27 Fig). The absence of internal coherence in their differences (reflected in their dispersed distribution) and of additional information to support the biological origin of these differences implied that this subset of samples was unlikely to reflect a biologically meaningful population structure. Since these samples dominated many of our preliminary results, we were concerned that the latter might be methodological artifacts. We, therefore, decided to eliminate these samples. This filter removed 28 samples.

As a result of this quality control, our final dataset is composed of 198 samples, including 51 samples of batch 1 (mean raw coverage of ~35x) and 147 samples from batch 3 (mean raw coverage of ~32x).

Application of the same filters to the initial set of 50 human samples from the Simons Genome Diversity Project [51], filtered out 18 samples (including HMM training samples), and ended up with a final set of 32 human samples.

### Allele calling

As a result of the copy number calling, for each window in the genome, we had a collection of copy number intervals expressing with 99% confidence the copy number of all individuals in our sample in that window. We identified copy number alleles in each of these windows through an allele calling algorithm. We here distinguish between *individual copy number intervals* and *allele copy number intervals*. The former refers to the copy number of each individual in a given window, while the latter refers to the copy number of a group of individuals sharing the same allele in a given window. The algorithm was applied individually to all windows as follows (see S28 Fig for a cartoon case example for clarification):

1. It identified the most common integer copy number among all individual copy number intervals of the window (in case of a tie between two or more integers, the lowest integer is chosen).
2. All the samples having this most common copy number(s) in their individual copy number intervals were classified as belonging to the major copy number allele.
3. An allele copy number interval was assigned to the major copy number allele. This interval contained all integer copy numbers shared by 90% or more of the samples already classified as belonging to the major allele.
4. All the samples whose individual copy number interval overlaps with the major copy number allele interval were classified as belonging to the major copy number allele.
5. Once the major allele was identified, the same procedure (steps 1 to 4) was repeated iteratively with the remaining samples (if any) to identify the second, third, and successively most frequent alleles until no samples were left.

This algorithm aims to retrieve the different alleles present in each sample. The windows that showed more than one allele were classified as being CNV windows. They were classified as CNV gains when containing copy number alleles with a copy number higher than diploid, as CNV losses when there was a loss of copy number (allele copy number with less than the diploid copy number) or as CNV gains and losses when there were alleles with copy numbers both higher and lower than the diploid copy number.

This allele-calling algorithm groups most of the samples into the major allele, and was consequently very conservative in calling more than one allele. Only samples with a 99% copy number interval clearly differentiable from the main allele were classified as having the minor allele. For the windows with a single allele, we first classified as diploid all the windows whose copy number allele interval contained the copy number 2. Then, we classified as FD regions all the windows in which none of the individual sample intervals spanned more than three copy numbers. Finally, we classified as unclassified regions all the other noisy windows for which we could not confidently distinguish more than one allele but where the intervals were not narrow.

After classifying each non-diploid window in a given category, we grouped consecutive windows of the same category into longer non-diploid regions of a given category. Of note, these non-diploid regions might be the result of more than one duplication or deletion event as consecutive windows in the same category could have a different duplication/deletion history. Moreover, our resolution was limited by the 1 kbp of non-consecutive windows. For this reason, with this approach, we were blind to differences in copy number within these windows. In practice, these differences appear as noise of the copy number estimates.

### Comparison to aCGH in rhesus macaque

To assess our genome-wide copy number maps, we used a previously published microarray-based comparative genomic hybridization (aCGH) data of 10 macaque individuals [36]. From a total of 123 CNV regions reported by Lee et al. [36] (on autosomal chromosomes), 63 (51.2%) survived the conversion from rheMac2 to rheMac8 (liftOver). We report 29 (46%) of these 63 regions as non-diploid regions. Moreover, we report 18 (28.6%) of these 63 regions as variable in copy number. We recover a significant part of the regions detected as CNV by Lee et al. [36] with a completely different technique and approach. They characterize fewer samples (10), use a much older assembly (rheMac2), and have a much lower resolution (~4 kbp defined by 6 consecutive 50–75 nt long probes). Private CNV cases and differences in the approach account for the part of Lee et al. [36] CNV regions that we do not recover (54%). As our call of variability in non-duplicated regions is designed to be very conservative, it is not surprising that part of Lee et al. [36] CNV regions appear as fixed duplications or unclassified regions in our classification. Nevertheless, still, a big part of them are reported as CNV in our analysis.

### Comparison to other human copy number maps

Copy number calls in the human genome were compared to the annotation of segmental duplications in the hg19 human reference genome [55] and the CNV calls in humans from Sudmant et al. [63].

We merged overlapping annotations of segmental duplications in order to avoid redundant counts. On the one hand, 75.52% of the segmentally duplicated regions in the human genome overlapped with at least one of our non-diploid regions. Our copy number calls were more stringent than the segmental duplication calls regarding the identity between copies needed to be detected. For this reason, we did not retrieve part (24.48%) of the segmentally duplicated regions. On the other hand, 57.03% of our non-diploid windows overlapped with a segmentally duplicated region. The rest (42.97%) might not overlap because of two reasons. First, segmental duplications are genome-assembly dependent. If a given duplication (CNV or not) is not present (or resolved) in the assembly, it will not be annotated as a segmental duplication. Second, segmental duplications are defined as regions that share 90% or more identity with another region of the assembly for 1 kbp or more length. With the read-depth based copy number calls, we were sensitive to smaller identity tracks.

The comparison with CNV calls in Sudmant et al. [63] is not ideal. First, in Sudmant et al. [63], CNV calls were performed with the 1,000 Genomes Project phase 3 WGS data, including 2,504 individuals from 26 populations. This means a sample size considerably bigger than the 32 human samples that we used to perform human maps. Second, in Sudmant et al. [63], CNV calls were performed per population in order to see the within-population copy number variability. To compare rhesus macaque copy number maps with copy number variability present in human populations, we designed our human sample set to detect extensive inter-population variability. Despite that, we detected as non-diploid 27.81% and saw variation in 5.39% of Sudmant et al. [63] CNV calls. If we only consider CNV regions larger than 5,000 bp, percentages increase up to 41.44% and 9.45%.

### Duplicated genes by age and segmental duplications

We compared our estimated chromosomal content of all types of non-diploid windows with independent proxies: duplicated genes and the segmental duplications annotated in the rhesus macaque genome. We used data of precise phylostratification of rhesus macaque duplicated genes [56] to classify duplicated genes by three different age categories: first, *macaque specific genes* only present in the macaque species; second, *other primate genes* present in other primates but not only in macaques; and third, *other mammalian duplicated genes* found also duplicated in rhesus macaque that appeared before the primate diversification within the mammal class. For segmental duplications annotations, we used rheMac2 annotations from the UCSC table browser.

### Definition of genes implicated in FD regions, CNV regions or unclassified regions

We used Ensembl (release 92) annotations of rhesus macaque (rheMac8) protein-coding genes and a list of reliable protein-coding genes in the human genome from Abascal et al. [62]. For those genes with more than one protein-coding transcript annotated in Ensembl (release 92), we selected only the exons of the transcripts used in the Ensembl Compara multi-species database (the main isoform). Genes with more than one annotated protein-coding transcript and no annotation in Ensembl Compara multi-species were not considered. According to these criteria, we considered 20,946 rhesus macaque protein-coding genes and 17,000 human protein-coding genes. The difference in the number of the considered protein-coding genes reflects the differences in the quality of the annotations of these two species reference genomes.

We divided the considered genes into several categories according to their copy number profile to identify the ones with copy number differences between human and rhesus macaque. First, we considered the genes that had part of their protein-coding region (at least 1 bp) overlapping with a non-diploid window. According to this, we considered genes that were overlapping with FD regions, with CNV regions, and with unclassified regions. Among these genes, we considered those that had 90% or more of their protein-coding region within FD regions or CNV regions as being within these regions. Among the genes within FD regions or CNV regions, we searched for complete FD and complete CNV genes. To be considered a complete FD gene or a complete CNV gene, at least half of the samples had to have a constant pattern of copy number along the gene length. This means that the copy number intervals in all the windows covering the gene had to contain the same copy number (at least one integer copy number must be shared by all the intervals) in at least half of the samples. We then ran the allele calling algorithm for the whole gene in order to identify the possible gene alleles. Genes with more than one gene allele were considered complete CNV genes and genes with only one gene allele were considered complete FD genes.

### Comparison of rhesus macaque and human gene copy number profiles

We extracted the list of rhesus-human orthologous genes from Ensembl (release 92). We considered those orthologous pairs that were within both the list of considered rhesus macaque protein-coding genes and the list of considered human ones.

We crossed the gene calls for rhesus macaque with the gene calls for their orthologs in humans. Moreover, we took human copy number information from Sudmant et al. [63] and the annotation of human segmental duplications in hg19 [55].

### Disease association exploration

The GWAS Catalog [64] was parsed in order to retrieve genes of interest with variants associated with a complex disease or phenotype (downloaded 2018/09/26). We recovered the associations considering both Reported genes (gene names reported by the paper) and the Mapped genes (mapped gene given the position of the SNV).

In order to obtain even more possible associations between genes and diseases, we used the DisGeNET tool [65] that compiles information from several public databases and uses the power of text mining to find links between genes and diseases in the literature.

## Supporting information

S1 Table List and metadata of samples. (XLSX)

S2 Table List of non-diploid regions in the rhesus macaque genome. (XLSX)

S3 Table Rhesus macaque and human orthologous gene pairs copy number profile. (XLSX)

S1 Fig Summary of the methodology. (A) Starting with NGS data and a masked genome reference, we followed a series of steps in order to call copy number intervals for each non-repetitive 1 kbp window and sample (see Methods). We performed a copy number allele calling per window to classify all non-diploid windows in FD regions, CNV regions or unclassified non-diploid regions. (B) To assess the functional implications of these non-repetitive windows, we crossed the protein-coding genes in the corresponding genome with the three types of non-diploid windows (see Methods). From all the genes that were related to non-diploid windows, we distinguished between those overlapping with the three different categories of non-diploid windows. Among those genes overlapping with FD regions, we focused on those that had 90% or more of their coding region duplicated and fixed and, among these, we focused on the ones that were complete FD genes (see Methods). Equally, among those genes overlapping with CNV regions, we focused on those genes within CNV regions (90% or more of the coding region) and those that were complete CNV genes. The same pipeline was applied to both the rhesus macaque and the human set of samples.
(EPS)

S2 Fig Landscape of the non-diploid regions in the human reference genome. Genome-wide map of human non-diploid regions. Chromosomes are represented by horizontal bars. FD regions, CNV gains, CNV losses, CNV gain/losses, and unclassified regions are represented as color-coded marks. Sex chromosomes, centromeric and telomeric regions are not included.
(EPS)

S3 Fig Chromosomal contribution of non-diploid regions and duplicated genes in the rhesus macaque reference genome. (A) Proportion of the length covered by FD regions, CNV gains, CNV losses, CNV gain/losses, unclassified regions per chromosome. (B) Proportion of the length covered by segmental duplications, rhesus macaque specific genes, other primate genes and other mammal genes per chromosome.
(EPS)

S4 Fig Proportion of the length covered by FD regions, CNV gains, CNV losses, CNV gain/losses, unclassified regions per chromosome in the human reference genome. (EPS)

S5 Fig Schematic example of our gene classification according to its copy number context. Top: schematic representation of a protein-coding gene (exons shown as black boxes) over the track of repetitive regions (in purple) that are masked from the reference genome before mapping (see Methods). Panels A-H: three consecutive non-repetitive 1 kbp windows covering the length of the gene are represented in all the panels. 99% confidence copy number intervals for 5 samples are depicted in color transitions (plus a black and white pattern for the diploid copy number) as examples. All the copy number intervals present in each window are listed below each case. The 8 gene copy number relationships considered are exemplified (A-H). First, the gene will be classified as being diploid if all of its protein-coding length is covered by diploid windows (A). Second, the gene will be classified as partial FD (B), partial CNV (E) or non-diploid unclassified gene (H) if it partially overlaps with the respective type of non-diploid region. Third, the gene will be considered composed FD gene (C) or composed CNV gene (F) if 90% or more of its coding length is covered by the corresponding type of non-diploid region having a non-constant number of copies per sample along its protein-coding sequence length. Finally, the gene will be classified as being complete FD gene (D) or complete CNV gene (G) if there is a constant copy number per sample along all its protein-coding region (see Methods).
(EPS)

S6 Fig Gene length and percentage of its coding region overlapping with FD regions, CNV regions and other unclassified non-diploid regions in rhesus macaque. (A) Gene length distribution of all the considered genes. (B) Mean percentage of the coding region overlapping with non-diploid regions (FD regions, CNV regions, and unclassified regions) for each gene length decile. (C) Distribution of genes that overlap with the three types of non-diploid regions with respect to the percentage of their coding regions that overlaps. (D) Mean gene length for each decile depending on the percentage of the coding region overlapping with non-diploid regions. Results are shown for each non-diploid region type.
(EPS)

S7 Fig Copy number profile of all orthologous protein-coding genes in rhesus macaque and human. (A and B) Venn diagrams representing, first, the entire set of orthologous protein-coding genes in each species; second, the number of genes overlapping with FD regions (blue), CNV regions (orange) and unclassified regions (grey) in open circles; third, genes within FD regions (blue) and CNV regions (orange) light-colored circles; and fourth, complete FD genes (blue) and complete CNV genes (orange) in dark-colored circles. All areas are approximations. (C) Colored table representing the overlap in diploid, complete FD genes and complete CNV genes of orthologous genes from each species. (D) Colored table representing the number of human genes with orthologs in rhesus macaque intersecting with human segmental duplications and Sudmant et al. (2015) human CNV calls. Only diploid, complete FD and complete CNV macaque genes were considered in this summarized version of the table. See S9 Fig for the complete version of these tables.
(EPS)

S8 Fig Copy number profile comparison of orthologous pairs between human and rhesus macaque. All the considered protein-coding genes in the rhesus macaque genome that have an annotated orthologous pair were considered. Numbers correspond to the number of orthologous pairs in each of the represented categories: (A) diploid genes, genes overlapping with unclassified regions, genes overlapping with FD regions, complete FD gene, genes overlapping with CNV regions, complete CNV gene, (B) genes within Sudmant et al. (2015) CNV calls and within segmental duplications.
(EPS)

S9 Fig Copy number profile comparison of one-to-one orthologous pairs between human and rhesus macaque. One-to-one human-rhesus macaque orthologous pairs were considered. Numbers correspond to the number of orthologous pairs in each of the represented categories: (A) diploid genes, genes overlapping with unclassified regions, genes overlapping with FD regions, complete FD gene, genes overlapping with CNV regions, complete CNV gene, (B) genes within Sudmant et al. (2015) CNV calls and within segmental duplications.
(EPS)

S10 Fig Copy number along the sequence of ILF2 in rhesus macaques (top) and in humans (bottom). The X axis represents the genomic sequence and the Y axis represents the distribution of copy number across all our samples in each window. Vertical dashed lines represent window limits, horizontal black line at the bottom represents the corresponding gene length and black boxes represent exons. The copy number interval of each individual in each window is represented as a light blue box encompassing the span of its copy number interval in each window or a thick line in case of copy number integers. All individuals are superposed in the image: darker shades of green imply more individuals with the given number of copies of the given window.
(EPS)

S11 Fig Copy number along the sequence of SYCP3 in rhesus macaques (top) and in humans (bottom). The X axis represents the genomic sequence and the Y axis represents the distribution of copy number across all our samples in each window. Vertical dashed lines represent window limits, horizontal black line at the bottom represents the corresponding gene length and black boxes represent exons. The copy number interval of each individual in each window is represented as a light blue box encompassing the span of its copy number interval in each window or a thick line in case of copy number integers. All individuals are superposed in the image: darker shades of green imply more individuals with the given number of copies of the given window.
(EPS)

S12 Fig Copy number along the sequence of HSPB8 in rhesus macaques (top) and in humans (bottom). The X axis represents the genomic sequence and the Y axis represents the distribution of copy number across all our samples in each window. Vertical dashed lines represent window limits, horizontal black line at the bottom represents the corresponding gene length and black boxes represent exons. The copy number interval of each individual in each window is represented as a light blue box encompassing the span of its copy number interval in each window or a thick line in case of copy number integers. All individuals are superposed in the image: darker shades of green imply more individuals with the given number of copies of the given window.
(EPS)

S13 Fig Copy number along the sequence of PVRIG in rhesus macaques (top) and in humans (bottom). The X axis represents the genomic sequence and the Y axis represents the distribution of copy number across all our samples in each window. Vertical dashed lines represent window limits, horizontal black line at the bottom represents the corresponding gene length and black boxes represent exons. The copy number interval of each individual in each window is represented as a light blue box encompassing the span of its copy number interval in each window or a thick line in case of copy number integers. All individuals are superposed in the image: darker shades of green imply more individuals with the given number of copies of the given window.
(EPS)

S14 Fig Copy number along the sequence of APOL2 in rhesus macaques (top) and in humans (bottom). The X axis represents the genomic sequence and the Y axis represents the distribution of copy number across all our samples in each window. Vertical dashed lines represent window limits, horizontal black line at the bottom represents the corresponding gene length and black boxes represent exons. The copy number interval of each individual in each window is represented as a light blue box encompassing the span of its copy number interval in each window or a thick line in case of copy number integers. All individuals are superposed in the image: darker shades of green imply more individuals with the given number of copies of the given window.
(EPS)

S15 Fig Copy number along the sequence of SPDYE4 in rhesus macaques (top) and in humans (bottom). The X axis represents the genomic sequence and the Y axis represents the distribution of copy number across all our samples in each window. Vertical dashed lines represent window limits, horizontal black line at the bottom represents the corresponding gene length and black boxes represent exons. The copy number interval of each individual in each window is represented as a light blue box encompassing the span of its copy number interval in each window or a thick line in case of copy number integers. All individuals are superposed in the image: darker shades of green imply more individuals with the given number of copies of the given window.
(EPS)

S16 Fig Copy number along the sequence of VAPB in rhesus macaques (top) and in humans (bottom). The X axis represents the genomic sequence and the Y axis represents the distribution of copy number across all our samples in each window. Vertical dashed lines represent window limits, horizontal black line at the bottom represents the corresponding gene length and black boxes represent exons. The copy number interval of each individual in each window is represented as a light blue box encompassing the span of its copy number interval in each window or a thick line in case of copy number integers. All individuals are superposed in the image: darker shades of green imply more individuals with the given number of copies of the given window.
(EPS)

S17 Fig Copy number along the sequence of PPP2R5C in rhesus macaques (top) and in humans (bottom). The X axis represents the genomic sequence and the Y axis represents the distribution of copy number across all our samples in each window. Vertical dashed lines represent window limits, horizontal black line at the bottom represents the corresponding gene length and black boxes represent exons. The copy number interval of each individual in each window is represented as a light blue box encompassing the span of its copy number interval in each window or a thick line in case of copy number integers. All individuals are superposed in the image: darker shades of green imply more individuals with the given number of copies of the given window.
(EPS)

S18 Fig Copy number along the sequence of MPC2 in rhesus macaques (top) and in humans (bottom). The X axis represents the genomic sequence and the Y axis represents the distribution of copy number across all our samples in each window. Vertical dashed lines represent window limits, horizontal black line at the bottom represents the corresponding gene length and black boxes represent exons. The copy number interval of each individual in each window is represented as a light blue box encompassing the span of its copy number interval in each window or a thick line in case of copy number integers. All individuals are superposed in the image: darker shades of green imply more individuals with the given number of copies of the given window.
(EPS)

S19 Fig Copy number along the sequence of HSD17B7 in rhesus macaques (top) and in humans (bottom). The X axis represents the genomic sequence and the Y axis represents the distribution of copy number across all our samples in each window. Vertical dashed lines represent window limits, horizontal black line at the bottom represents the corresponding gene length and black boxes represent exons. The copy number interval of each individual in each window is represented as a light blue box encompassing the span of its copy number interval in each window or a thick line in case of copy number integers. All individuals are superposed in the image: darker shades of green imply more individuals with the given number of copies of the given window.
(EPS)

S20 Fig Copy number along the sequence of CCDC115 in rhesus macaques (top) and in humans (bottom). The X axis represents the genomic sequence and the Y axis represents the distribution of copy number across all our samples in each window. Vertical dashed lines represent window limits, horizontal black line at the bottom represents the corresponding gene length and black boxes represent exons. The copy number interval of each individual in each window is represented as a light blue box encompassing the span of its copy number interval in each window or a thick line in case of copy number integers. All individuals are superposed in the image: darker shades of green imply more individuals with the given number of copies of the given window.
(EPS)

S21 Fig Copy number along the sequence of JUND in rhesus macaques (top) and in humans (bottom). The X axis represents the genomic sequence and the Y axis represents the distribution of copy number across all our samples in each window. Vertical dashed lines represent window limits, horizontal black line at the bottom represents the corresponding gene length and black boxes represent exons. The copy number interval of each individual in each window is represented as a light blue box encompassing the span of its copy number interval in each window or a thick line in case of copy number integers. All individuals are superposed in the image: darker shades of green imply more individuals with the given number of copies of the given window.
(EPS)

S22 Fig Copy number along the sequence of NAT8 in rhesus macaques (top) and in humans (bottom). The X axis represents the genomic sequence and the Y axis represents the distribution of copy number across all our samples in each window. Vertical dashed lines represent window limits, horizontal black line at the bottom represents the corresponding gene length and black boxes represent exons. The copy number interval of each individual in each window is represented as a light blue box encompassing the span of its copy number interval in each window or a thick line in case of copy number integers. All individuals are superposed in the image: darker shades of green imply more individuals with the given number of copies of the given window.
(EPS)

S23 Fig Copy number along the sequence of ENY2 in rhesus macaques (top) and in humans (bottom). The X axis represents the genomic sequence and the Y axis represents the distribution of copy number across all our samples in each window. Vertical dashed lines represent window limits, horizontal black line at the bottom represents the corresponding gene length and black boxes represent exons. The copy number interval of each individual in each window is represented as a light blue box encompassing the span of its copy number interval in each window or a thick line in case of copy number integers. All individuals are superposed in the image: darker shades of green imply more individuals with the given number of copies of the given window.
(EPS)

S24 Fig Copy number along the sequence of HIST1H2AC in rhesus macaques (top) and in humans (bottom). The X axis represents the genomic sequence and the Y axis represents the distribution of copy number across all our samples in each window. Vertical dashed lines represent window limits, horizontal black line at the bottom represents the corresponding gene length and black boxes represent exons. The copy number interval of each individual in each window is represented as a light blue box encompassing the span of its copy number interval in each window or a thick line in case of copy number integers. All individuals are superposed in the image: darker shades of green imply more individuals with the given number of copies of the given window.
(EPS)

S25 Fig Copy number along the sequence of TXN in rhesus macaques (top) and in humans (bottom). The X axis represents the genomic sequence and the Y axis represents the distribution of copy number across all our samples in each window. Vertical dashed lines represent window limits, horizontal black line at the bottom represents the corresponding gene length and black boxes represent exons. The copy number interval of each individual in each window is represented as a light blue box encompassing the span of its copy number interval in each window or a thick line in case of copy number integers. All individuals are superposed in the image: darker shades of green imply more individuals with the given number of copies of the given window.
(EPS)

S26 Fig Quality control and filtering of samples. (A and B) Scatter plots representing the standard deviation of the coverage in diploid control regions (X axis), the Kolmogorov-Smirnov statistic for normality of the distribution of coverage (Y axis in A) and the Pearson correlation coefficient between neighboring windows (Y axis in B) for each sample. Dashed lines represent the thresholds for each of the three parameters (0.5 for the standard deviation of the coverage in diploid control regions; 0.03 for the Kolmogorov-Smirnov statistic; and 0.15 for the Pearson correlation coefficient). (C) Box plots representing the number of Mbp (mega base pairs) of non-diploid regions for each batch of data. We were overcalling non-diploid regions in batch 2 (with lower mean coverage in all samples) and decided to exclude it. (D) First two principal components of a PCA of the genome-wide copy number estimates. Color represents the raw average genome-wide coverage. A group of lower average coverage samples appears separated from the rest. We decided to impose a threshold of 11.7 average raw coverage that excludes all the separated samples. (E) First two principal components of a PCA with the remaining samples. Color indicates the origin of individual samples: Chinese (yellow), Indian (red), or Indian/Chinese hybrids (orange). Black circle indicates the major cluster of samples selected as the final set of samples.
(EPS)

S27 Fig First two principal components of a principal component analysis performed with single-nucleotide variants. (PNG)

S28 Fig Toy example representing the allele-calling procedure. Supposing we had 30 samples with copy number intervals for a given window as shown in the figure, this image shows what would be the result of our allele-calling procedure. Samples are ordered *a posteriori*. The steps would be the following, in accordance with the steps detailed in the Allele calling Methods section:
1. The most common integer copy number among all samples is copy number 4 (20 out of 30 samples).
2. Samples 1 to 20 are classified as Allele 1.
3. The allele copy number interval assigned to Allele 1 is 3–4 since 90% (18/20) of the Allele 1 samples share this interval. This interval did not include copy number 2 or copy number 5 because samples including these integers in their copy number intervals represent less than 90% of the Allele 1 group.
4. Sample 21 was included in the Allele 1 group since its copy number interval (2–3) overlaps with the copy number interval assigned to Allele 1 (3–4).
5. The same procedure (steps 1 to 4) is carried out with the remaining 9 samples. First, copy number 2 (diploid) is detected as the most common integer copy number. Samples 22 to 27 are classified as Allele 2. The copy number interval assigned to Allele 2 is 2 since samples with copy number 1 do not add up to 90% of the Allele 2 group. No additional sample is included in the Allele 2 group.
6. The same procedure (steps 1 to 4) is carried out with the remaining 3 samples. Integer copy numbers 5 and 6 are the most common, but 5 is chosen since it is the lowest number. Samples 28 to 30 are classified as Allele 3. The copy number interval assigned to Allele 3 is 5–6 since more than 90% of the Allele 3 samples share this interval.
7. No samples are left to be classified.
Summing up, our algorithm classifies our 30 samples in three alleles: samples 1 to 21 belong to Allele 1 with an allele copy number interval of 3–4, samples 22 to 27 belong to Allele 2 with an allele copy number interval of 2 (diploid), and samples 28 to 30 belong to Allele 3 with an allele copy number interval of 5–6. This window would be classified (further down the pipeline) as copy number variant.
(EPS)
