# Supplementary figures and images for: Copy number variants and fixed duplications among 198 rhesus macaques (Macaca mulatta)
Source: PLoS Genet. 2020 May 11;16(5):e1008742. doi: 10.1371/journal.pgen.1008742 (PMC7241854; doi:10.1371/journal.pgen.1008742)

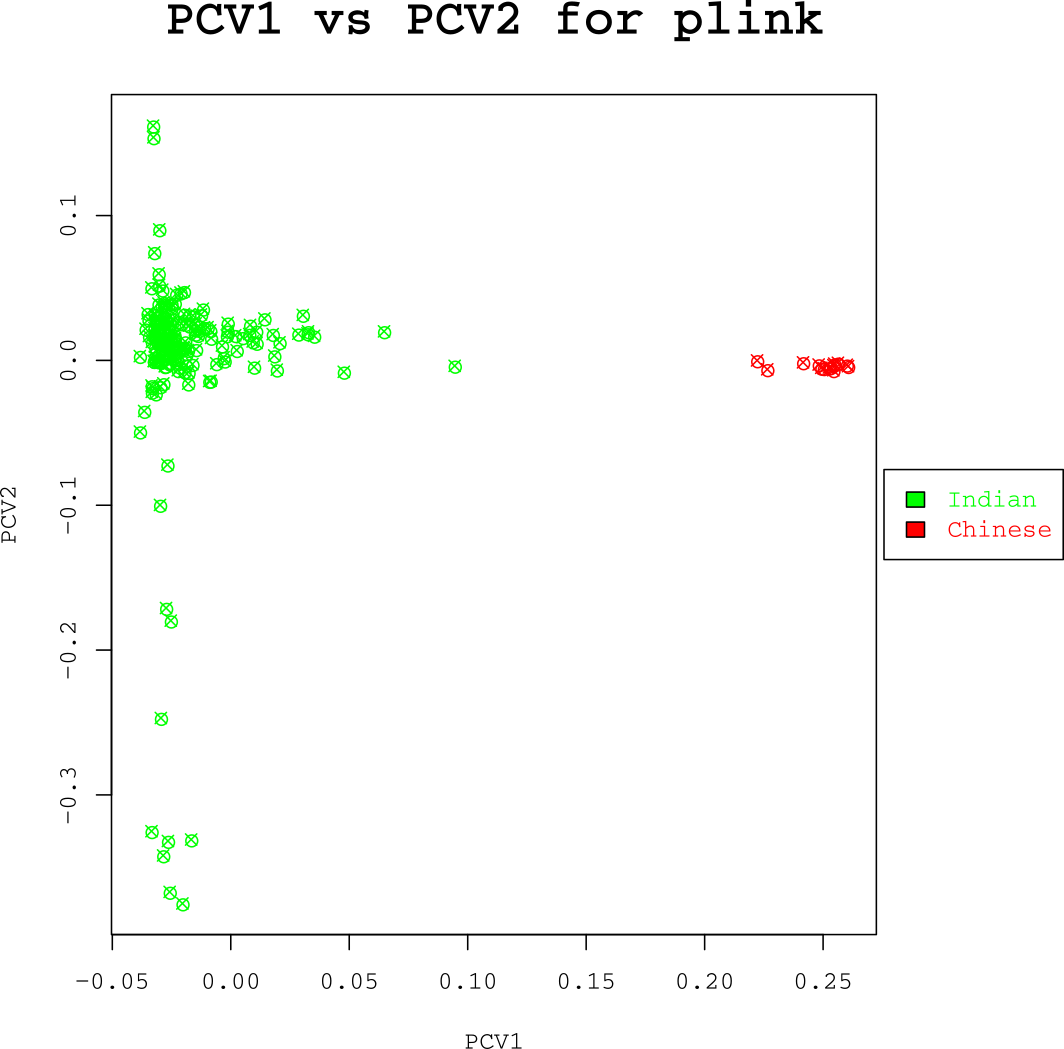

Supplement: S27 Fig — (PNG) [file pgen.1008742.s030.png]
